# Bixafen Induces Programmed Cell Death in *Rhizoctonia solani* by Damaging Mitochondrial Integrity

**DOI:** 10.3390/jof12040238

**Published:** 2026-03-26

**Authors:** Yuanhang Ren, Ping Huang, Wentao Gu, Ruyi Li, Yongtian Zhao, Lidan Lu

**Affiliations:** 1 Key Laboratory of Coarse Cereal Processing, Ministry of Agriculture and Rural Affairs, Sichuan Engineering and Technology Research Center of Coarse Cereal Industralization, College of Food and Biological Engineering, Chengdu University, Chengdu 610106, China; renyuanhang@cdu.edu.cn (Y.R.);; 2 School of Life Science and Agriculture, Qiannan Normal University for Nationalities, Duyun 558000, China

**Keywords:** bixafen, *Rhizoctonia solani*, mitochondria, apoptosis, autophagy

## Abstract

Rice sheath blight caused by *Rhizoctonia solani* is one of the most destructive diseases of rice. Bixafen has been proposed as a promising control agent with moderate resistance risk; however, its cellular mode of action remains unclear. Therefore, this study investigated the antifungal mechanism of bixafen from the perspective of programmed cell death (PCD). Bioassays showed that bixafen strongly inhibited *R. solani*, with a median effective concentration (EC_50_) of 1.16 μg/mL. Morphologically, bixafen induced hyphae collapse, vacuolization, chromatin aggregation, and mitochondrial disruption. Transcriptome analysis further revealed that bixafen significantly altered the expression of genes involved in the tricarboxylic acid cycle and PCD pathways. In addition, bixafen, at the concentration of EC_50_, triggered ROS accumulation accompanied by increased malondialdehyde (MDA) levels. These oxidative effects led to mitochondrial damage, characterized by loss of membrane potential, reduced Tomm20 expression, and decreased Aco-2 activity. Subsequently, bixafen activated apoptosis, as evidenced by induction of the mitochondria-associated inducer of death (AMID), down-regulation of Bcl-2, and DNA fragmentation. Moreover, bixafen also induced autophagy by reducing p62 and increasing Beclin-1 expression, which suggests the clearance of damaged mitochondria. Collectively, these results demonstrated that bixafen induced mitochondrial-dependent apoptosis and autophagy in *R. solani*, which provided novel insights into its cellular antifungal mechanism and supported its potential as a PCD-targeted fungicide.

## 1. Introduction

Rice ranks among the world’s three most important staple crops, feeding over half of the global population. However, its production is severely threatened by rice sheath blight, a widespread disease caused by *Rhizoctonia solani* [1]. This pathogen attacks plants at all growth stages, causing annual yield losses of 30% in major rice growing regions [2]. At present, the deployment of resistant varieties remains the long-term approach for managing rice sheath blight. However, continuous variation in *R. solani* physiological races often breaks down host resistance, which has become a major constraint in achieving durable control through genetic resistance [3]. Consequently, chemical control is widely regarded as the most direct and effective strategy for disease suppression.

Many fungicides have been registered to control rice sheath blight in China, such as demethylation inhibitors (DMIs), succinate dehydrogenase inhibitors (SDHIs), or quinone outside inhibitors (QoIs) [4]. However, due to extensive and repetitive applications of these fungicides over a long period of time, the field populations of *R. solani* have developed different degrees of resistance to various fungicides with single-site action, including tebuconazole, epoxiconazole, Jinggangmycin, and azoxystrobin [5]. For instance, the overexpression of 14 α-demethylase formed by CYP51 genes is the main reason for *R. solani*’s resistance to two DMI fungicides, hexaconazole and difenoconazole [6]. Furthermore, the resistance of *R. solani* to thifluzamide has also been documented, which was initially registered in China in 1999 for sheath blight control [7]. Thifluzamide’s mechanism involves binding to succinate dehydrogenase (SDH), thereby disrupting the fungal energy metabolism. However, prolonged and extensive application of thifluzamide has intensified the selection of target-site mutants. Recent reports indicate that more than twenty fungal species associated with rice sheath blight have developed over eighty documented mutations in relevant genes, highlighting a growing resistance challenge [8]. The growing issue of fungicide resistance not only diminishes its control efficacy but also exerts a negative influence on public health and the environment. Thus, it is crucial to find some new fungicides.

Bixafen, an SDHI, targets succinate dehydrogenase, developed by Bayer Crop Science. The succinate dehydrogenase is a key enzyme common to both the mitochondrial electron transport chain and the tricarboxylic acid (TCA) cycle [9]. This fungicide has shown effectiveness against multiple fungal diseases, such as corn rust, gray leaf spot, wheat rust, and glume blotch. Moreover, bixafen holds potential for controlling potato early blight, particularly in cases where resistance to conventional fungicides has emerged [10]. Up to now, bixafen has not been registered for controlling rice sheath blight, and its underlying mechanism of action on *R. solani*, especially at the cellular level, is still uncertain.

Apoptosis and autophagy constitute two principal types of programmed cell death (PCD), each activated by diverse intracellular and environmental signals under precise regulatory mechanisms [11]. In recent years, a few studies have found that fungicides can induce programmed cell death in fungal hyphae. For example, Schuster et al. [12] reported that triazole’s fungicide lethality was due to a combination of reactive oxygen species-induced apoptosis and macroautophagy in the wheat pathogen *Zymoseptoria tritici* and the rice blast fungus *Magnaporthe oryzae*. Simultaneous inhibition of both programmed cell death pathways abolishes azole-induced cell death [12]. In addition, studies have demonstrated that lawsone suppresses *Fusarium* infection by inducing autophagy-related pathways, whereas magnolol promotes autophagosome formation in *Botrytis cinerea* and upregulates the expression of autophagy-associated genes, ultimately attenuating fungal pathogenicity [13,14]. Consequently, inducing fungal PCD represents a promising strategy for pathogen control. This approach may not only constitute a novel antifungal mechanism but may also help mitigate resistance development and inform the design of next-generation therapeutics.

In this study, bixafen was observed to trigger oxidative stress and subsequent mitochondrial dysfunction, initiating a co-regulatory pathway involving damaged mitochondria that culminated in apoptosis, autophagy, and hyphae death. These results confirmed that bixafen functioned as a PCD-inducing agent, capable of activating both apoptotic and autophagic processes in hyphae, providing new insights for the antifungal mechanism of bixafen at the cellular level.

## 2. Materials and Methods

### 2.1. Materials and Reagents

The *R. solani* strain used in this study was provided by Qiannan Normal University for Nationalities and maintained on Potato Dextrose Agar (PDA) at 28 °C. Bixafen (purity ≥ 98%) was sourced from Bepharm Science & Technology Co., Ltd. (Shanghai, China). The DCFH-DA dyes, malondialdehyde (MDA) assay kit, and BCA protein quantification kit were procured from Keygen Biotech Co., Ltd. (Nanjing, China). Fluorescent probes including JC-1, DAPI, propidium iodide (PI), Hoechst 33342, and mito-tracker were supplied by Beyotime Biotechnology (Beijing, China). Primary antibodies against GAPDH (1:2000), p62 (1:1000), Tomm20 (1:1000), Aco-2 (1:1000), mitochondria-associated inducer of death (AMID) (1:1000), Beclin-1 (1:1000), and Bcl-2 (1:1000), as well as HRP-conjugated secondary antibody (1:5000), were purchased from Servicebio Co., Ltd. (Wuhan, China).

### 2.2. Antifungal Bioassay In Vitro

The antifungal activity of bixafen against *R. solani* was evaluated using the hyphae growth rate method [15]. Bixafen was dissolved in DMSO and incorporated into high-pressure sterilized PDA medium (cooled to approximately 50 °C) to achieve final concentrations of 0.25, 0.5, 1, 2, and 4 μg/mL. The DMSO-only (final concentration of 1%) treatment served as the blank control. Hyphae disks (0.7 cm in diameter) of *R. solani* were inoculated onto PDA plates and incubated at 28 °C in darkness. When the control group had grown to cover two-thirds of the plate diameter, colony diameters were measured with the cross method. The hyphae growth inhibition rate was calculated using a standard formula.

$$\mathrm{Inhibition}\left(\%\right)=\frac{\mathrm{Cd}\left(\mathrm{cm}\right)\;-\;\mathrm{Td}\left(\mathrm{cm}\right)}{\mathrm{Cd}\left(\mathrm{cm}\right)\;-\;0.7\left(\mathrm{cm}\right)}\;\times \;100$$

Cd (cm) is the average colony diameter of the control group, and Td (cm) is the average colony diameter of the bixafen treatment group.

### 2.3. Hyphae Morphology Observations by Scanning and Transmission Electron Microscopy

The hyphae disks of *R. solani* were transferred to Potato Dextrose Broth (PDB) and cultured at 28 °C with shaking at 160 rpm for 48 h. After 24 h, the hyphae were treated with bixafen at final concentrations of 1.2 μg/mL (EC_50_) and 7.6 μg/mL (EC_75_). The DMSO (final concentration at 1%) served as the blank control. The harvested hyphae were dehydrated in absolute ethanol for 45 min, followed by critical point drying with liquid carbon dioxide. Subsequently, the samples were mounted on scanning electron microscopy (SEM) stubs, sputter coated with gold (60 s, 1.8 mA, 2.4 kV) in an SC7620 system (Polaron, London, UK), and imaged under a JSM-6510LV scanning electron microscope (JEOL, Tokyo, Japan).

Hyphae ultrastructure was analyzed using transmission electron microscopy (TEM) (HT7700, Hitachi, Tokyo, Japan) according to standard procedures. Samples were fixed in 2.5% glutaraldehyde in phosphate-buffered saline, post-fixed with 1% osmium tetroxide, and dehydrated through a graded ethanol series. Following propylene oxide treatment and Epon Polybed 812 embedding, ultrathin sections were stained with uranyl acetate and lead citrate for TEM observation [1].

### 2.4. RNA-Seq Analysis

Hyphae were prepared as described in Section 2.3. Total RNA was isolated from *R. solani* using TRIzol reagent (Invitrogen, Carlsbad, CA, USA) for transcriptomic analysis. Sequencing libraries were constructed following Illumina standard protocols and sequenced in paired-end mode on an Illumina NovaSeq 6000 platform. Raw reads were filtered using Fastp (v0.19.3), and clean reads were aligned to the reference genome (NCBI ID: 1557640) with HISAT2 (v2.1.0) under default settings. Gene expression levels were quantified as FPKM (fragments per kilobase per million mapped reads). Differential expression analysis was performed with EdgeR (v3.12.1) in R, applying thresholds of |log_2_FC| ≥ 1 and adjusted * *p* < 0.05 to identify significant DEGs. Functional enrichment of DEGs was analyzed using Goatools-v0.6.10 for GO terms and KOBAS-v3.0 for KEGG pathways [16].

### 2.5. ROS Detection and MDA Content

Hyphae were prepared as outlined in Section 2.3. After washing twice with PBS, the samples were incubated with 10 μM DCFH-DA in the dark at 28 °C for 30 min, then washed and resuspended in PBS. Fluorescence intensity was measured at an excitation wavelength of 525 nm. Hyphae homogenate was prepared by weighing 100 mg of hyphae, freeze grinding, and processing sequentially by ultrasonication (400 W, 1 s intervals, 10 min) and centrifugation (10,000 rpm, 4 °C, 10 min). The resulting supernatant was analyzed for MDA content according to the manufacturer’s protocol [17].

### 2.6. Measurement of Mitochondrial Membrane Potential (MMP) Assay

Hyphae prepared according to Section 2.3 were collected, washed three times with PBS, and stained with 1 μM JC-1 in the dark at 28 °C for 30 min. Mitochondrial membrane potential was assessed by fluorescence microscopy (Leica TCS SP8, Wetzlar, Germany) using excitation/emission wavelengths of 488 nm and 620 nm [18].

### 2.7. Mitochondria Quantity

Mitochondria quantity in hyphae was quantified via staining with mito-tracker dye. Hyphae, prepared as described in Section 2.3, were washed three times with PBS and incubated with 1 μM of probe in the dark at 28 °C for 30 min. Fluorescence was immediately observed under a microscope (Leica TCS SP8, Leica Microsystems, Wetzlar, Germany) using excitation/emission settings of 488/540 nm [19].

### 2.8. Programmed Cell Death Assay

Hyphae viability was evaluated by dual staining with Hoechst 33342 and PI. Hyphae prepared as in Section 2.3 were washed three times with PBS and incubated with 10 µg/mL Hoechst 33342 and 5 µg/mL PI in the dark at 28 °C for 30 min. Samples were then observed under a fluorescence microscope using excitation/emission wavelengths of 350/461 nm for Hoechst 33342 and 535/617 nm for PI [15]. Nuclear morphology and DNA fragmentation in hyphae were evaluated by dual staining with DAPI and TUNEL. Following the treatment described above, TUNEL staining was performed according to the manufacturer’s protocol. Briefly, hyphae were incubated with terminal deoxynucleotidyl transferase (TdT) and FITC-labeled dUTP in the dark at 28 °C for 60 min, then counterstained with 1 µg/mL DAPI for 10 min at room temperature. Samples were observed under a fluorescence microscope using excitation/emission settings of 488/550 nm for FITC and 405/475 nm for DAPI [15].

### 2.9. Western Blotting

Hyphae, prepared as described in Section 2.3, were harvested and lysed in ice-cold RIPA buffer, and protein concentrations were determined using a BCA assay kit (Keygen Biotech, Nanjing, China). Immunoblotting was performed following established protocols with antibodies against Aco-2, Tomm20, p62, AMID, Beclin-1, and Bcl-2. Protein band intensities were quantified using Quantity One software v19.0 (Bio-Rad, Hercules, CA, USA), using GAPDH as the loading control [20,21].

### 2.10. Statistical Analysis

All experiments were conducted in triplicate. Statistical analyses were performed with SPSS 26.0 (IBM Corp., Armonk, NY, USA). Mean comparisons were carried out using Tukey’s Honestly Significant Difference (HSD) test or Student’s *t*-test, with significance defined as *p* < 0.05. Figures were generated using Origin Pro 2018 (OriginLab Corp., Northampton, MA, USA).

## 3. Result and Discussion

### 3.1. Antifungal Activity of Bixafen Against R. solani

**Figure 1:**
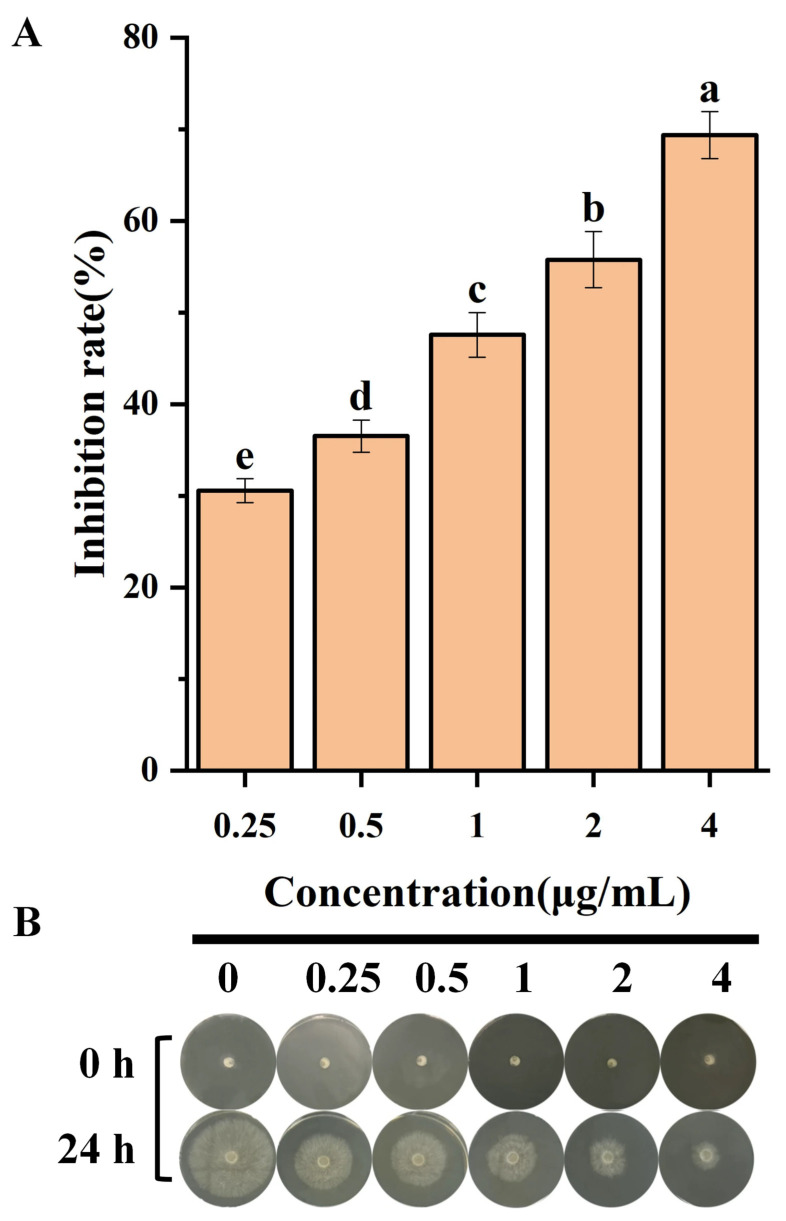
Inhibition rate (**A**) and hyphae growth status (**B**) of bixafen against *R. solani*. Different letters indicate significant differences (*p* ≤ 0.05) via Tukey’s HSD test analysis.

As shown in Figure 1A,B, bixafen demonstrated potent dose-dependent fungicidal activity against *R. solani*. The inhibition rate of bixafen against *R. solani* at 4 μg/mL is 66.84%. The EC_50_, EC_75,_ and EC_90_ values were 1.16 μg/mL, 7.58 μg/mL, and 49.36 μg/mL, respectively, with a toxicity regression equation of Y = 1.35X − 0.08 (Table 1). Bixafen has demonstrated efficacy against various fungal diseases, including corn rust, the early blight of potato, gray leaf spot as well as rust, and the glume blotch of wheat [10]. Recent studies have validated bixafen efficacy against several pathogens on rice, such as *R. solani* and *Magnaporthe oryzae*. For example, Chen et al. found that the average EC_50_ value of bixafen against 100 wild-type strains of *M. oryzae* was 1.67 μg/mL [9]. Liu [22] reported that bixafen can inhibit the hyphae growth and sclerotia production of *R. solani.* Moreover, the field control effect of the 20% bixafen–pyrazole ether mixed suspension on *R. solani* could reach 69% [22]. *R. solani* is the primary causal agent of rice sheath blight, a disease that significantly reduces both grain yield and quality, resulting in substantial economic losses. At present, chemical fungicides remain an effective strategy for managing sheath blight and safeguarding rice production. Moreover, according to the Fungicide Resistance Action Committee (FRAC), bixafen are considered to be medium-to-high risk fungicides; therefore, they may have great prospects for future applications in rice sheath blight control [23].

### 3.2. Effects of Bixafen on Morphology and Ultrastructure of R. solani

**Figure 2:**
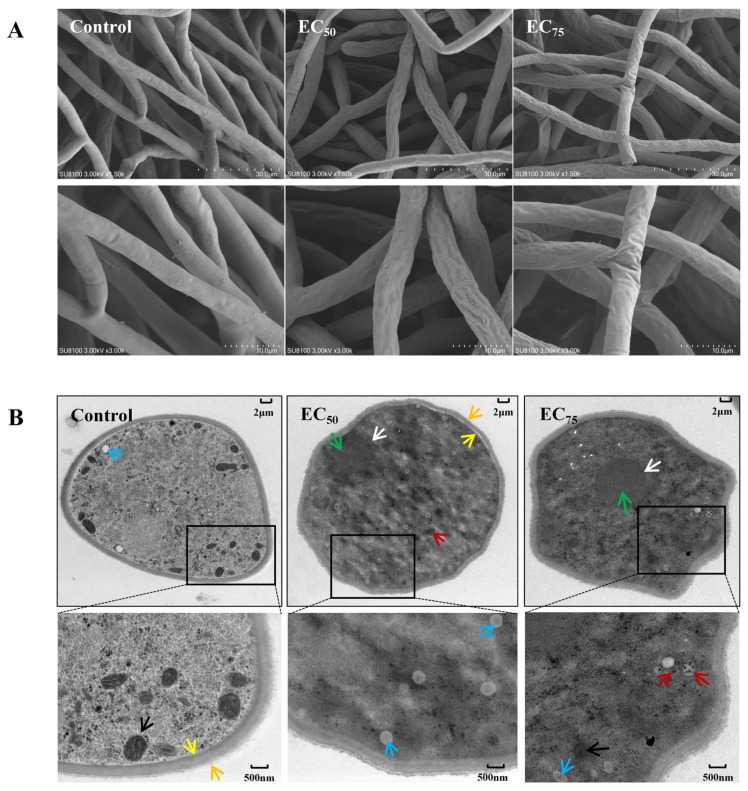
Observation of the ultrastructure of hyphae by SEM (**A**) and TEM (**B**) after bixafen treatment. Cell membrane (yellow arrow), cell wall (orange arrow), nucleus (white arrow), chromatin (green arrow), mitochondria (black arrow), vacuole (blue arrow), and autophagosome (red arrow).

Bixafen treatment induced hyphae dehydration and shrinkage, resulting in visible surface depression compared to the control (Figure 2A). At the EC_75_ concentration, the damage was more pronounced, with evident collapse and folding of the hyphae. TEM further revealed distinct ultrastructural changes in bixafen-treated hyphae (Figure 2B). Untreated hyphae displayed smooth surfaces, evenly distributed cytoplasm, and dense mitochondrial structures. In contrast, the EC_50_ treatment induced increased intracellular vacuolation, uneven cytoplasmic distribution, and plasmolysis. At the EC_75_ concentration, dissolved mitochondria, nuclear chromatin condensation, autophagic vesicles, and cell wall surface wrinkling were observed, which were all consistent with features of apoptosis and autophagy.

### 3.3. Transcriptome Analysis

**Figure 3:**
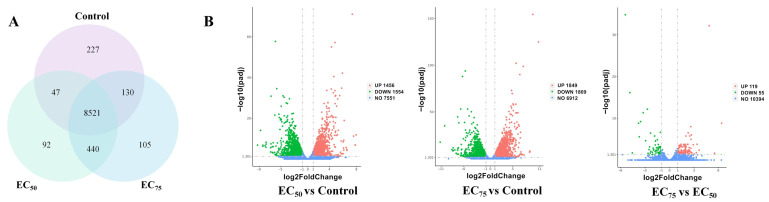
Differentially expressed genes in control and bixafen-treated groups shown with a Venn diagram (**A**) and volcano plot (**B**).

**Figure 4:**
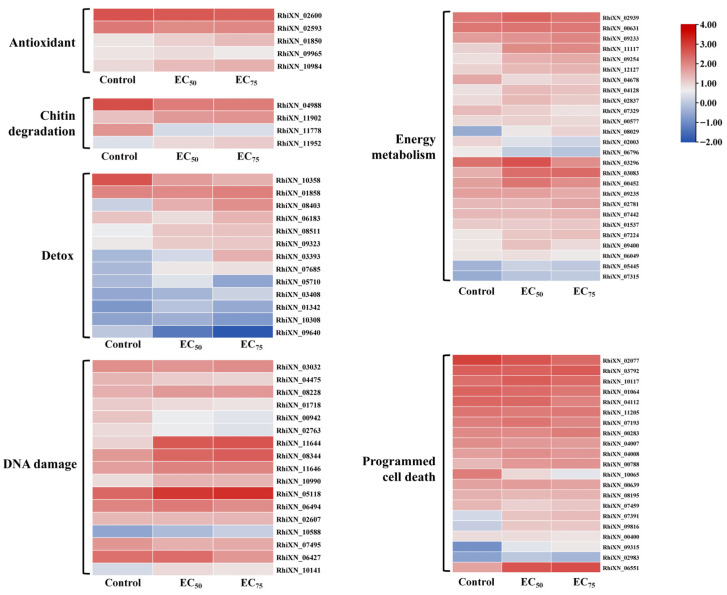
Heatmap of the differentially expressed genes in different functional categories.

More than 60 Gb of clean data were obtained with a Q30 score above 90% and over 80% of reads mapped to the reference genome, confirming data quality and suitability for downstream analysis. As shown in Figure 3A, a total of 9562 genes had been identified. Among them, the 3019 DEGs (1465 up-regulated and 1554 down-regulated), 3658 DEGs (1849 up-regulated and 1809 down-regulated), and 174 DEGs (119 up-regulated and 55 down-regulated) were obtained in the EC_50_ vs. control, EC_75_ vs. control, and EC_75_ vs. EC_50_ group, respectively (Figure 3B). Based on the functional annotation results, differentially expressed genes were classified into six categories including detox, antioxidant, chitin degradation, programmed cell death, DNA damage, and energy metabolism (Figure 4). In the “PCD” classification, most gene expression levels were up-regulated with an increasing concentration of bixafen. For example, compared to the control group, treatment with bixafen made the ferroptosis-related gene *RhiXN_06551* (membrane iron permease) increase significantly by 63% (EC_50_) and 69% (EC_75_), respectively. Bixafen treatment resulted in the up-regulation of *RhiXN_00639* (serine/threonine protein kinase), *RhiXN_02077* (heat shock protein HSP70), and *RhiXN_08195* (apoptosis-inducing factor), respectively. In addition, genes in the “Antioxidant” and “Energy metabolism” category, such as *RhiXN_02593* (glutathione S-transferase), *RhiXN_06796* (ATPase), and *RhiXN_03296* (citrate synthase), were significantly down-regulated in the bixafen treatment group. In the “DNA damage” group, the gene *RhiXN_02763* involved in DNA repair showed a significant decrease, while the treatment group was down-regulated by 33% (EC_50_) and 51.5% (EC_75_), respectively, after treatment with bixafen.

**Figure 5:**
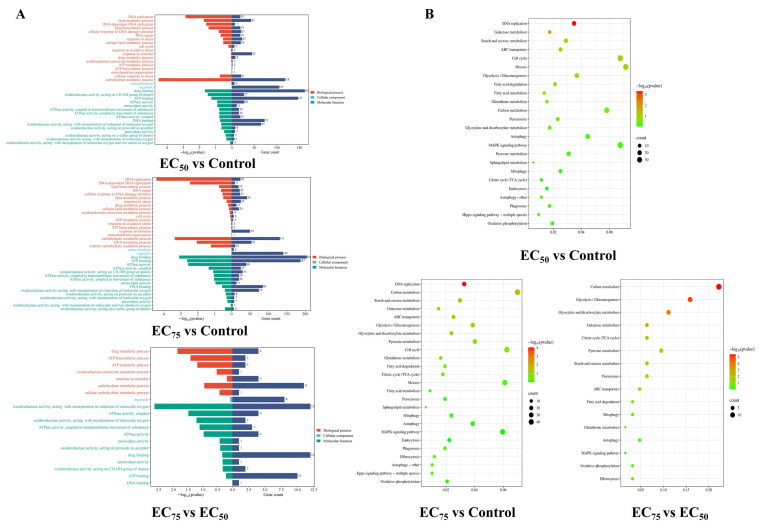
Gene ontology (**A**) and Kyoto Encyclopedia of Genes and Genomes (**B**) enrichment analysis for differentially expressed genes.

As shown in Figure 5A, treatment with bixafen leads to the “DNA replication”, “carbohydrate metabolic process”, “ATPase activity”, and “Oxidoreductase activity” terms being enriched in GO analysis. The results indicated that bixafen has a significant interference with intracellular substance DNA and ATP synthesis. Meanwhile, antioxidant enzyme activity-related terms were enriched, indicating an imbalance in the intracellular reactive oxygen species environment. KEGG enrichment analysis yielded similar findings. Pathways related to energy metabolism and biosynthesis, such as “DNA replication”, “carbon metabolism”, “Pyruvate metabolism” and “MAPK signaling”, were significantly enriched after bixafen treatment, reflecting the broad activation of energy synthesis routes under stress. Furthermore, the programmed cell death pathways, “Autophagy” and “Mitophagy”, were also enriched in response to bixafen (Figure 5B). The morphological and transcriptomic data presented above suggested that bixafen induced mitochondrial dysfunction in hyphae. This hypothesis was further examined through biochemical assays.

### 3.4. Mitochondrial Damage

**Figure 6:**
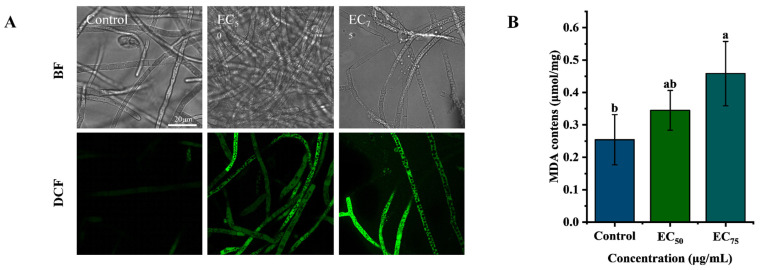
The effects of bixafen on oxidative stress in hyphae. Analysis of ROS accumulation by epifluorescence (**A**). The content of MDA (**B**). Different letters indicate significant differences (*p* ≤ 0.05) via Tukey’s HSD test analysis.

**Figure 7:**
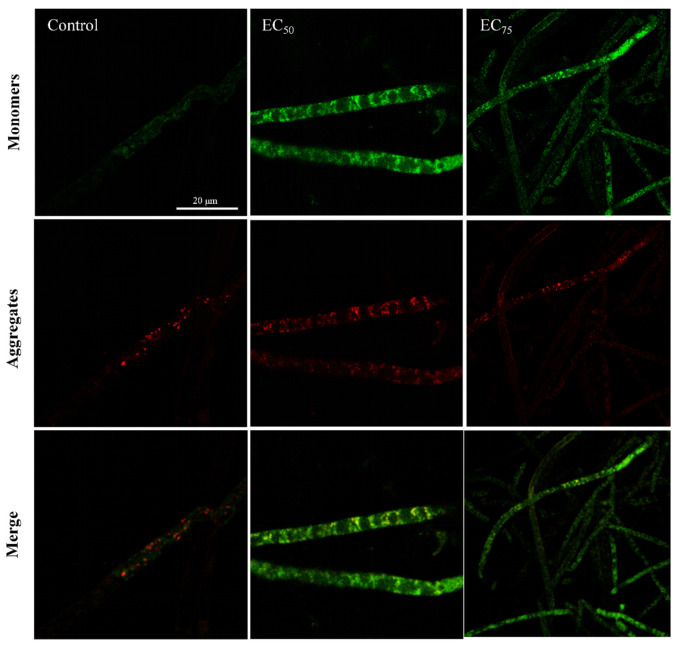
The analysis of bixafen on MMP loss in hyphae by epifluorescence.

It is well known that SDHI interferes with respiration by targeting the SDH complex. SDH is an essential and evolutionarily conserved mitochondrial enzymatic complex critically required for the proper functioning of both the electron transport chain and TCA cycle, both essential for cellular energy production and ATP synthesis [23]. Recent reports indicate that, in addition to energy deficits, these SDHIs cause multiple mitochondria damage-related adverse effects such as oxidative stress and apoptosis [24]. Mitochondria are essential cellular organelles that not only serve as the primary hub for energy metabolism but also play key roles in regulating physiological processes such as apoptosis and autophagy [25]. In our study, mitochondrial damage and dysfunction were found after treatment with bixafen. As shown in Figure 6A, green fluorescence intensity (DCF positive cells) increased in a concentration-dependent manner in bixafen-treated samples compared to the control, indicating ROS production in *R. solani*. The MDA, a marker of lipid peroxidation and oxidative stress, also rose dose-dependently in treated hyphae, reaching levels 1.4- and 1.8-fold higher than the control (Figure 6B). These results demonstrated that bixafen triggered ROS accumulation and lipid peroxidation in *R. solani*. Mitochondria are both a major source and a primary target of ROS [26]. With increasing bixafen concentrations, hyphae cells exhibited diminishing red fluorescence and a marked rise in green fluorescence (Figure 7), indicating MMP collapse in *R. solani*.

**Figure 8:**
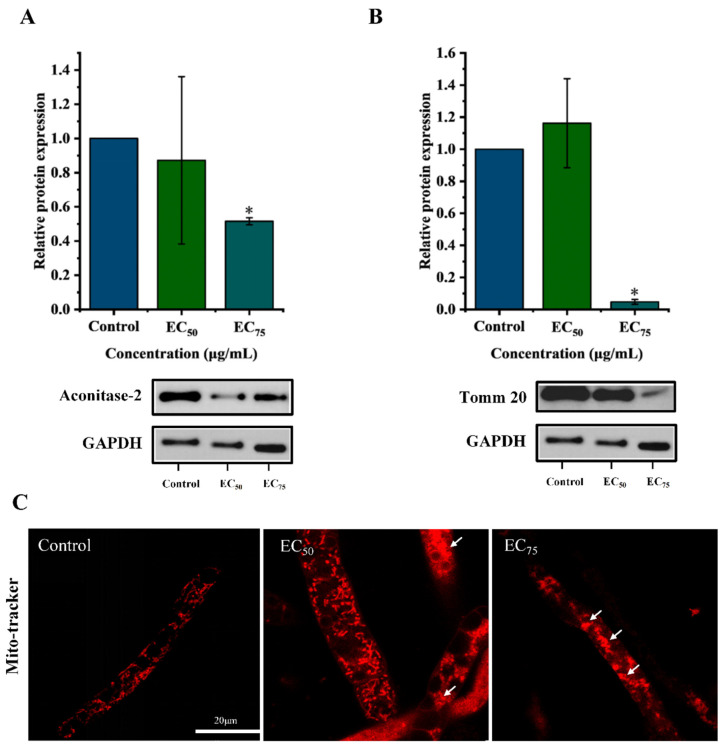
The effect of bixafen on mitochondrial damage. Expression level of Aco-2 (**A**) and Tomm 20 (**B**). The quantity of mitochondria (**C**). The white arrow indicates damaged mitochondria. The * indicates significant differences (*p* ≤ 0.05) versus control via Student’s *t*-test analysis.

To further verify mitochondrial structural and functional damage, the expression of two mitochondrial-associated proteins such as Aco-2 and Tomm20 was assessed by Western blot. As shown in Figure 8A,B, bixafen down-regulated both Aco-2 and Tomm20 in a dose-dependent manner. At the EC_75_ concentration, expression levels of Aco-2 and Tomm20 decreased by 48% and 95%, respectively. Aco-2 is a key enzyme in the TCA cycle that catalyzes the reversible conversion of citrate to isocitrate via the intermediate cis-aconitate, involving successive dehydration and rehydration steps [27]. As a critical bridge in the TCA cycle, Aco-2 links citrate entry to downstream energy-producing reactions and is essential for sustaining normal cellular metabolism and ATP synthesis. Tomm20 is a mitochondrial outer membrane protein and a component of the translocase complex. Previous studies have shown that exogenous agents can modulate mitochondrial membrane potential and ATP synthesis via Tomm20 [28,29]. These findings indicate that ROS overload subsequently induced mitochondrial impairment, marked by the reduced expression of Aco-2 and Tomm20 and decline of MMP.

The fluorescent probe mito-tracker was used to evaluate mitochondrial quantity. The results showed that after bixafen treatment at the EC_75_ concentration, the chiseled red fluorescence dots were significantly reduced, and the boundaries of the fluorescent dots were unclear and fused with each other (Figure 8C). The results suggested that bixafen treatment reduces the number of mitochondria in hyphal cells and induced mitochondrial swelling and degradation.

### 3.5. Programmed Cell Death

Apoptosis and autophagy represent two classical forms of PCD in eukaryotes [30]. As fundamental and tightly regulated cellular events, they both play essential roles in maintaining organismal homeostasis. However, excessive apoptosis and autophagy caused by drugs can interfere with the normal physiological activities of cells. For example, it was reported that bifonazole inhibited the respiratory chain in *Penicillium expansum* hyphae, resulting in markedly elevated levels of H_2_O_2_ and MDA. This oxidative burst leads to hyphal cell membrane damage and ultimately triggers both apoptosis and necroptosis in cells [31]. Anacardic acid induced typical apoptotic hallmarks in *M. oryzae*, including phosphatidylserine externalization, chromatin condensation, and DNA fragmentation. The compound was shown to inhibit *M. oryzae* spore germination and hyphae growth by triggering apoptosis [32].

**Figure 9:**
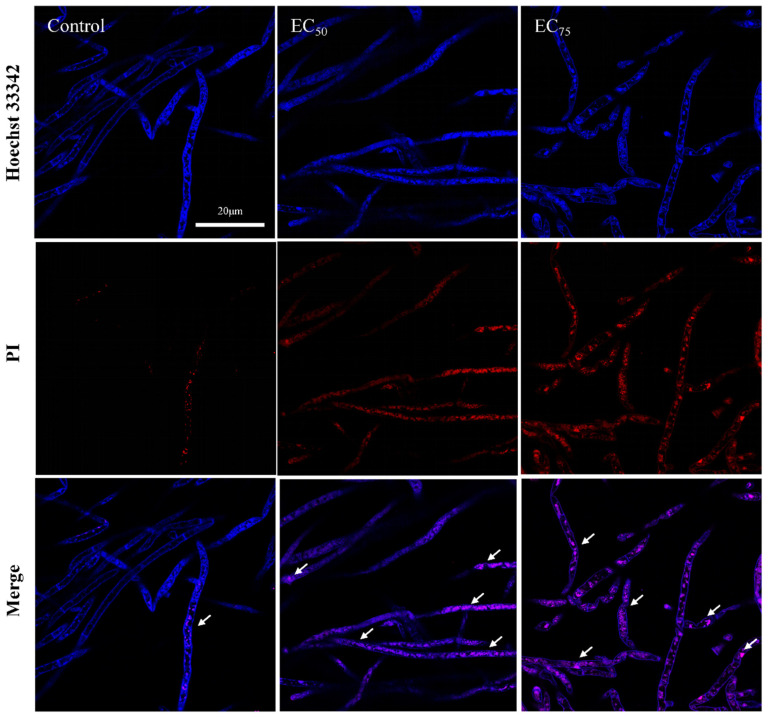
The analysis of bixafen on chromatin condensation by epifluorescence. The white arrow indicates a condensed chromatin mass.

**Figure 10:**
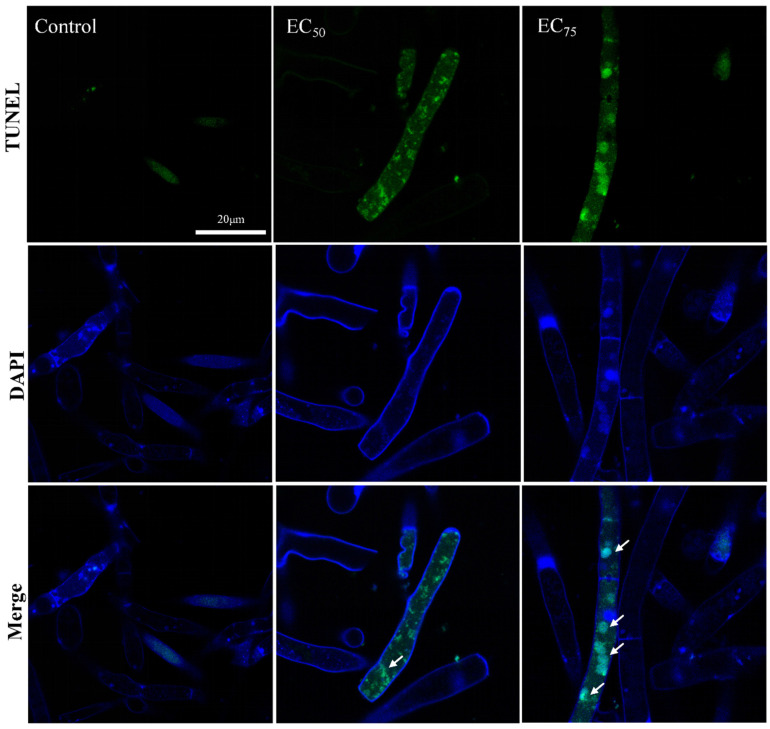
The analysis of bixafen on DNA breakage by epifluorescence. The white arrow indicates the site of DNA damage in the nucleus.

The apoptotic phenotype observed in yeast and filamentous fungi closely resembles that of mammals, characterized by DNA fragmentation, phosphatidylserine externalization, intracellular ROS accumulation, chromatin condensation, and nuclear marginalization [33,34]. As shown in Figure 9, Hoechst 33342 staining revealed brighter and more clustered blue fluorescence in bixafen-treated hyphal cells, indicating chromatin condensation induced by bixafen [35]. DNA damage was assessed using the TUNEL assay, which detects DNA strand breaks in apoptotic cells. Figure 10 shows enhanced green fluorescence with an increasing bixafen concentration, indicating aggravated DNA damage in *R. solani*. Chromatin condensation, mainly attributed to apoptotic signals, activates nucleases and structural rearrangement mechanisms within the nucleus, transforming chromatin from a loose state to a highly compact block-like structure. For instance, caspase-3 directly or indirectly activates the nuclease DNase, leading to DNA cleavage at internucleosomal sites and the formation of nucleosomal fragments [36]. In addition, AIF (apoptosis-inducing factor) and EndoG could also transfer from the mitochondria to the nucleus, degrading chromosomal DNA [37]. This DNA fragmentation or damage promotes chromatin relaxation and facilitates its subsequent reassembly [35]. Hence, the “DNA ladder” that DNA cleavage by caspase-3 was considered to be is a major feature of late apoptosis in mammals. However, this phenomenon has not been found in yeast, and the DNA fragmentation of filamentous fungi is also larger than that of mammals’ fragments of hundreds of bases [38].

**Figure 11:**
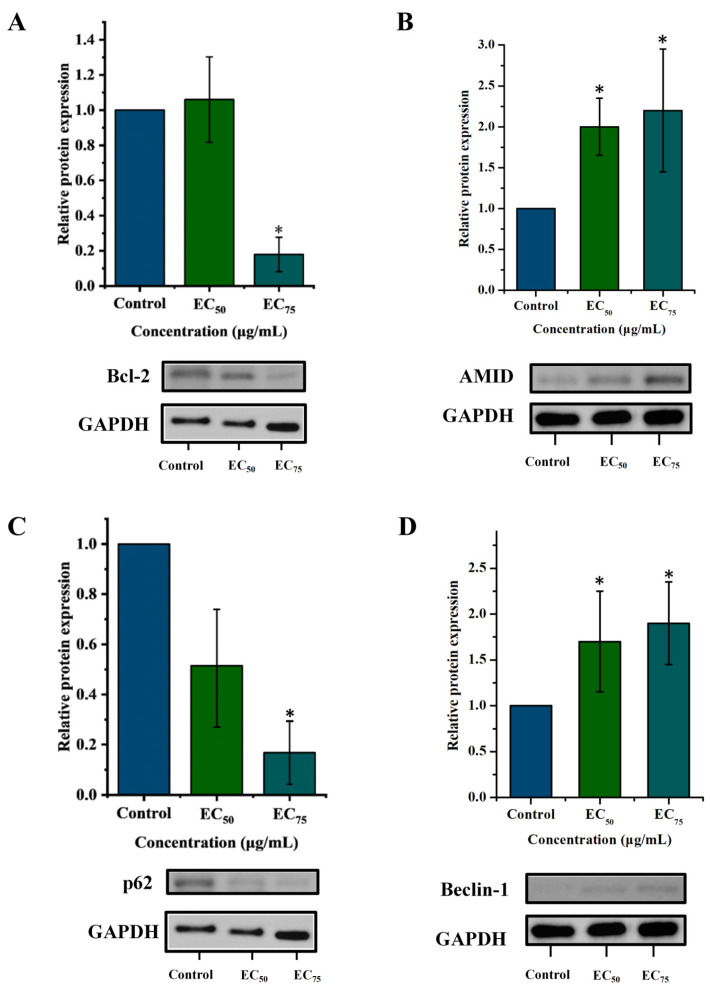
The effect of bixafen on apoptosis and autophagy-related protein expression levels of Bcl-2 (**A**), AMID (**B**), p62 (**C**), and Beclin-1 (**D**). The * indicates significant differences (*p* ≤ 0.05) versus control via Student’s *t*-test analysis.

Additionally, Western blot analysis revealed dose-dependent up-regulation of the AMID and down-regulation of the anti-apoptotic protein Bcl-2 by bixafen (Figure 11A,B). The AMID protein is a protein with sequence homology to the AIF family with NAD(P)H oxidase activity, which mainly participates in apoptosis in fungi by regulating the mitochondrial function and redox status [39]. The knockout or mutation of AMID homologous genes in *Neurospora crassa* and *Aspergillus nidulans* makes hyphae more unsensitive to apoptosis-inducing signals and ROS [36]. ROS accumulation up-regulates AMID protein expression, which in turn promotes further ROS generation. This positive feedback loop amplifies oxidative stress and accelerates the progression of apoptosis. Bcl-2, located on the mitochondrial outer membrane, stabilizes the mitochondrial membrane potential and inhibits cytochrome c (cyt-c) release, thereby suppressing apoptosis. In mammalian cells, cyt-c is recognized as an initial mitochondrial factor in the induction of apoptosis. Upon exposure to various pro-apoptotic signals, the mitochondrial permeability transition pore (MPTP) undergoes excessive and irreversible opening, resulting in the release of cyt-c, ultimately triggering apoptosis [40]. During this process, the pro-apoptotic protein Bax and the anti-apoptotic protein Bcl-2 regulate cyt-c release by forming homo- or heterodimeric pores on the mitochondrial membrane. In our study, we observed positive results for Bcl-2 and AMID, whose expression levels decreased or increased after the treatment of bixafen, respectively [41]. However, no bands of cyt-c or Bax were detected in this study. We speculated that it might be attributed to the monoclonal antibodies we used, which have low cross-reactivity to the corresponding *R. solani* proteins.

Autophagy is essential for degrading cytoplasmic proteins and organelles, including the selective clearance of damaged mitochondria to maintain mitochondrial quality and energy homeostasis [11]. Beclin-1, as a key component of the PI3K-III (class III phosphoinositide 3-kinase) complex, is an important biomarker for autophagic vesicle formation. Autophagic vesicle selectively recognizes proteins tagged with p62. This process is initiated by the binding of p62 to LC3 via its LIR (LC3-interacting region) domain, which facilitates the incorporation of ubiquitinated proteins into nascent autophagic vesicles. Following fusion with lysosomes to form autophagic vesicles, the acidification of the internal compartment leads to the complete degradation of p62 along with its associated cargo [42]. Western blot analysis confirmed that bixafen treatment increased Beclin-1 and reduced p62 expression compared to the control (Figure 11C,D), indicating autophagy induction. As with apoptosis-related proteins, antibody specificity also posed a challenge for autophagy markers. There were no bands corresponding to LC3-I or LC3-II detected. These limitations highlight the considerable gap between current research on fungal programmed cell death and the well-established framework in mammalian systems. In mammalian systems, biochemical techniques such as flow cytometry, Western blot, and immunohistochemistry are now routinely employed to investigate PCD pathways and their key regulators, particularly at the protein level. In contrast, research on the PCD of fungi, especially in filamentous fungi, remains constrained by several factors, including the presence of rigid cell walls, non-spore morphologies, and a lack of highly specific antibodies and fluorescent probes. These limitations restrict the experimental approaches available for confirming PCD in fungi, which currently rely heavily on morphological observations. Future efforts should focus on developing broader and more robust methods for detecting fungal PCD, thereby providing a foundation for deeper mechanistic studies into the regulatory factors and signaling pathways involved.

## 4. Conclusions

This study found that bixafen triggered oxidative stress, leading to mitochondrial dysfunction and the activation of a mitochondria-mediated cell death pathway in *R. solani*. These results provide new mechanistic insights into the antifungal activity of bixafen, which demonstrates that inducing endogenous fungal programmed cell death represents a promising strategy for pathogen control.

## Figures and Tables

**Table 1:** The activity of bixafen against *R. solani*.

Strain	EC50 (μg/mL)	95% Confidence Interval	EC75 (μg/mL)	95% Confidence Interval	EC90 (μg/mL)	95% Confidence Interval	Y = aX + b
*Rhizoctonia solani*	1.16	0.971–1.405	7.58	5.293–12.570	49.36	26.104–124.280	Y = 1.35X − 0.08

## Data Availability

The raw data supporting the conclusions of this article will be made available by the authors on request.
